# 

**Published:** 1858-01

**Authors:** 


NORTH AMERICAN

### MEDICO-CHIRURGICAL REVIEW.

Vol. II.] JANUARY, 1858. [No. 7.

### CONTENTS.

### ANALYTICAL AND CRITICAL REVIEWS.

| Art. | | Page |
|---|---|---|
| I.—1. | Elements of Pathological Anatomy. By S. D. Gross, M.D. | 1 |
| 2. | Rudiments of Pathological Anatomy. By Carl Wedl, M.D. | 1 |
| 3. | The Pathological Anatomy of the Human Body. By Julius Vogel, M.D. | 1 |
| 4. | Anatomical Description of the Diseases of the Organs of Circulation. By C. E. Hasse, M.D. | 1 |
| 5. | A Manual of Pathological Anatomy. By C. Rokitansky, M.D. | 2 |
| 6. | A Compendium of Pathological Anatomy. By A. W. Otto, M.D. | 2 |
| 7. | A Treatise on Pathological Anatomy. By G. Andral, M.D. | 2 |
| 8. | A Treatise on Pathological Anatomy. By W. E. Horner, M.D. | 2 |
| 9. | Histoire Anatomique des Inflammations. Par A. N. Gendrin, D.M., etc. | 2 |
| 10. | Histoire des Phlegmasies, etc. Par F. J. V. Broussais, D.M. | 2 |
| 11. | Essai sur l'Anatomie Pathologique. Par Jean Cruveilhier, D.M., etc. | 2 |
| 12. | Anatomie Pathologique. Par Laennec & Bayle (Dictionnaire de Médecine) | 2 |
| II.—1. | The Botanical Text-Book. By Asa Gray, M.D. | 22 |
| 2. | First Lessons in Botany. By the same author | 22 |
| 3. | Genera of the Plants of the United States. By the same author. | 22 |
| 4. | Manual of the Botany of the Northern United States. By the same author | 22 |
| III.— | The Cattle Plague and Diseased Meat, in their relations with the Public Health. By J. S. Gamgee | 35 |
| | A Second Letter on the same subject. By the same author | 35 |
| IV.— | A Catalogue of Preparations Illustrative of Diseases of the Ear. In the Museum of Joseph Toynbee, F.R.S., etc. | 42 |
| V.— | A New Description of the Entire Body (in Chinese.) By Benjamin Hobson, M.D. | 43 |
| VI.— | A Dictionary of Medical Science. By Robley Dunglison, M.D. | 44 |
| VII.— | General Therapeutics and Materia Medica. By Robley Dunglison, M.D. | 44 |

### ORIGINAL COMMUNICATIONS.

| | | |
|---|---|---|
| I.— | Some Remarks on Cataract. By C. S. Fenner, M.D. | 45 |
| II.— | Convulsions: their Pathology and Treatment. By B. M. Wible, M.D. | 56 |
| III.— | Adaptation of the Clamp and Button Sutures to Prolapse of the Vagina. By Horatio R. Storer, M.D. | 64 |
| IV.— | Three Cases of Infantile Apoplexy. By J. H. Wythes, M.D. | 70 |
| V.— | Congenital Hydro-Cerebral Hernia. By H. P. Ayres, M.D. | 73 |
| VI.— | Case of Fracture of the Skull and Wound of the Brain. By E. Wallace, M.D. | 75 |
| VII.— | Case of Luxation of the Knee-Joint. By C. Hixon, M.D. | 76 |
| VIII.— | Introduction of the Probang into the Larynx. By F. D. Leute, M.D. | 78 |

ART. PAGE

IX.—Plastic Operation upon the Ear. By J. PANCOAST, M.D. . . . 78
X.—Bony Tumor of the Scrotum. By J. G. KERR, M.D. . . . . 79
XI.—Hospitals of Paris. By RICHARD J. DUNGLISON, M.D. . . . . 81
XII.—Mortality of Philadelphia. By WILSON JEWELL, M.D. . . . . 86
XIII.—Proceedings of the Pathological Society of Philadelphia: Fatty Degeneration of Liver—Fistula of Gall-Bladder—Gangrene of Bladder—Encephaloid Tumor of Thigh—Primary Cancer of Pancreas—Bony Tumor of Scrotum—Cancer of Liver, and other Abdominal Viscera—Softening of Stomach—Fungus of Testicle —Injury of Shoulder-Joint—Ovary containing Bone . . . 93

AMERICAN PROGRESS OF MEDICAL SCIENCE.

Report on the Progress of Physiology and Anatomy. By S. WEIR MITCHELL, M.D. . . . . . . . . . . . . . . 105

BI-MONTHLY PERISCOPE.

ANATOMY AND PHYSIOLOGY.

1. Physiology of the Large Intestine . . . . . . . . . 131
2. Histology of the Nervous System . . . . . . . . . 133
3. Effect of Red Blood on the Vital Properties of the Contractile and Nervous Tissues . . . . . . . . . . . . 135
4. Structure of the Nervous Centres . . . . . . . . . 136

SURGERY.

1. Escharotic Treatment of Cancer . . . . . . . . . . 138
2. Excision of Joints . . . . . . . . . . . . 142
3. Excision of the Knee-Joint . . . . . . . . . . . 146
4. Operation for Imperforate Rectum . . . . . . . . . 151
5. Laceration of Urethra by Muscular Contraction . . . . . . 154
6. Ligature of Dorsalis Penis Artery for Hypertrophy of Prepuce . . 155
7. Removal of Warts by Chromic Acid . . . . . . . . . 156
8. Ligature of Common Carotid Artery in Epilepsy . . . . . . 157
9. Extraction of a Foreign Body from Œsophagus . . . . . . 158
10. Accidental Severance of Common Carotid Artery—Recovery . . 159
11. Large Uterine Polypus removed by the Ecraseur . . . . . . 160
12. Cancer and its Treatment . . . . . . . . . . . 161

MATERIA MEDICA AND THERAPEUTICS.

1. Action of Cod-liver Oil . . . . . . . . . . . . 162
2. Oil of Tansey . . . . . . . . . . . . . 165
3. Arsenic in Cholera . . . . . . . . . . . . 165

PATHOLOGY AND MEDICINE.

1. Dysentery a cause of Hepatic Abscess—Twenty-two Cases . . . 169
2. Secretion of Urinary Constituents from Skin . . . . . . 174
3. Intestinal Obstruction, with Operation . . . . . . . . 176

EDITORS' TABLE.—Quack Advertisements in Religious Newspapers. . 179
Solutions and Deposits of Mineral Springs. . . . . . . 186
Coffee in the Turkish Fashion. . . . . . . . . . 191
Jenner Monument. . . . . . . . . . . . . . 192
Biological Society of Philadelphia. . . . . . . . . . 193
New Physiological Journal by Brown-Séquard. . . . . . . 193
Cholera. . . . . . . . . . . . . . . . 194
Suit for Malpractice. . . . . . . . . . . . . 195
Pathology versus Phrenology . . . . . . . . . . 196
Prize Essays of the American Medical Association . . . . . 197

NECROLOGICAL NOTICES.—Dr. Abraham Stout. . . . . . . . 197
Dr. S. P. Hullihen. . . . . . . . . . . . . 199
Dr. W. H. Deadrick. . . . . . . . . . . . . 205
Dr. Samuel Metcalfe . . . . . . . . . . . . . 206
BI-MONTHLY BIBLIOGRAPHICAL RECORD . . . . . . . . 208

